# Case control study: magnetic resonance spectroscopy of brain in HIV infected patients

**DOI:** 10.1186/s12883-016-0628-x

**Published:** 2016-07-12

**Authors:** Devender Bairwa, Virendra Kumar, Surabhi Vyas, Bimal Kumar Das, Achal Kumar Srivastava, Ravinder M. Pandey, Surendra K. Sharma, Naranamangalam R. Jagannathan, Sanjeev Sinha

**Affiliations:** Department of Medicine, All India Institute of Medical Sciences, Ansari Nagar, New Delhi, 110029 India; Department of NMR & MRI Facility, All India Institute of Medical Sciences, Ansari Nagar, New Delhi, 110029 India; Department of Radio-diagnosis, All India Institute of Medical Sciences, Ansari Nagar, New Delhi, 110029 India; Department of Microbiology, All India Institute of Medical Sciences, Ansari Nagar, New Delhi, 110029 India; Department of Neurology, All India Institute of Medical Sciences, Ansari Nagar, New Delhi, 110029 India; Department of Biostatistics, All India Institute of Medical Sciences, Ansari Nagar, New Delhi, 110029 India

**Keywords:** Magnetic resonance spectroscopy, MRS, HIV, AIDS, Glutamate

## Abstract

**Background:**

In vivo proton magnetic resonance spectroscopy (^1^H-MRS) studies on brain in HIV infected patients have shown significant alteration in neuro-biochemicals.

**Methods:**

In this study, we measured the neuro-biochemical metabolites from the left frontal white matter (FWM) and left basal ganglia (BG) caudate head nucleus in 71 subjects that include 30 healthy controls, 20 asymptomatic HIV and 21 HIV patients with CNS lesion. Proton MR spectra were acquired at 3 T MRI system and the concentration (institutional units) of tNAA (N-acetylaspartate, NAA + N-acetylaspartylglutamate, NAAG), tCr (Creatine, Cr + phosphocreatine, PCr), choline containing compounds (tCho), glutamate + glutamine (Glx) and lipid and macromolecules at 0.9 ppm were determined using LC Model.

**Results:**

In BG, the concentration of tNAA (6.71 ± 0.64) was decreased and in FWM, the concentration of Glx (20.4 ± 7.8), tCr (9.14 ± 3.04) and lipid and macromolecules at 0.9 ppm (8.69 ± 2.96) were increased in HIV patients with CNS lesion. In healthy controls, the concentration of tNAA in BG was 7.31 ± 0.47 and concentration of Glx, tCr and lipid and macromolecules in FWM were 15.0 ± 6.06, 6.95 ± 2.56, 5.59 ± 1.56, respectively.

**Conclusion:**

Reduced tNAA in BG suggests neuronal loss in HIV patients with CNS lesion while increased Glx in FWM may suggest excito-toxicity. In addition, increased levels of tCr in FWM of HIV patients were observed. The study indicates region specific metabolic changes in tNAA, tCr and Glx in brain of HIV infected patients.

## Background

Human immunodeficiency virus (HIV) continues to be a major global public health issue. Approximately 36.9 [34.3–41.4] million people were living with HIV in 2014 whereas 2.0 [1.9-2.2] million people died of HIV related illnesses in 2014 and more than 95 % of all HIV-infected people now live in developing world [1]. India ranks third with the estimated number of people living with HIV/AIDS is approximately 2.089 million (2012) with an estimated adult (15–49 age group) HIV prevalence of 0.27 % in 2011 [2].

Central nervous system (CNS) involvement in HIV can occur both early as well as late. Early involvement is characterized by impaired abstracting ability, learning difficulties and slow speed of information processing. Late involvement of CNS can be either by neoplasms or opportunistic infections [3].

Opportunistic infection includes toxoplasmosis, cryptococcosis, progressive multifocal leucoencephalopathy and tuberculosis. Neoplasms include primary CNS lymphoma, Kaposi’s sarcoma and result of HIV-1 infection includes aseptic meningitis, HIV-associated neurocognitive impairment, including HIV encephalopathy/ AIDS dementia complex [4].

MRS studies of brain have reported change in metabolite levels in HIV patients. Many studies have reported changes in N-acetylasparatate (NAA) and creatine (Cr) levels in different regions of brain, however, the results reported across these studies are not consistent and sometimes contradictory [5–8]. Chong et al. [5] reported that there is significant reduction in the mean NAA to choline (Cho) ratio and NAA/Cr and an increase in Cho/Cr was observed in HIV patients with immunosupressed state and neurologic signs. Suwanwelaa et al. [7] showed a statistically significant reduction in NAA/Cr and NAA/Cho in centrum semiovale and thalamic areas with no statistically significant difference in Cho/Cr and mI/Cr in both the regions. The difference of NAA/Cr was more pronounced in white matter than in gray matter. In addition, HIV infection has been linked with neuro-cognitive impairment which has been shown to result in increased excite-toxicity of glutamate [9, 10]. In this context, glutamate concentration in brain has been studied and found to be significantly increased in CSF, plasma and brain tissues by using biochemical methods in patients with HIV infection [9]. In the present study, we have quantified changes in brain neuro-biochemical levels in patients with HIV infection at 3.0 Tesla using in vivo single voxel proton magnetic resonance spectroscopy (^1^H MRS).

## Methods

### Participants

A total of 71 subjects were recruited under this study from March 2014 to October 2015 including 30 healthy subjects (Group 1), 20 HIV asymptomatic patients (Group 2) and 21 HIV patients with CNS lesions (Group 3). Institute Ethics Committee approved the study and written informed consent was obtained from all the participants in the study. In case of patients with altered sensorium, consent was obtained from their relatives accompanying them to the hospital. Healthy subjects were with no history of high risk behavior, any prolonged medical or surgical illness.

### Physical examination and neurological evaluation

All the subjects underwent general physical examination and detailed clinical neurological examination. Subjects in Group 2 included sero-positive HIV patients with CD4 count <500/ul and on anti-retroviral therapy (ART), with normal neurological, neuropsychological examination and no apparent CNS involvement and were screened with the help of international HIV dementia scale for any possible subclinical HIV associated neuro-degeneration (HAND). Group 3 included HIV patient, with CNS involvement apparent on imaging (MR, CT, PET), blood investigations or CSF study. Socioeconomic status was derived by using Modified Kuppuswamy Scale last modified in 2012 [11]. For all participants baseline respiratory rate, heart rate, blood pressure and temperature were recorded.

### Biochemical investigations

HIV status was confirmed for all subjects using ELISA for HIV1 and HIV2 infection. In case of patients in Group 2 and Group 3, most recent CD4 count was recorded. Since neuro-biochemical levels are affected by dyslipidemia, diabetes and other derangement of normal physiology, laboratory parameters were obtained for all the participants. These included, complete blood counts, kidney function test, liver function test, fasting lipid profile and a fasting blood glucose level.

### Magnetic resonance imaging and spectroscopy

All MR studies were carried out on a whole body 3 Tesla MRI scanner (Achieva/Ingenia, Phillips Healthcare, The Netherlands). Routine MRI of brain including multi-slice T2-weighted axial images in three orthogonal planes and diffusion-weighted images acquired with a standard head coil. T2-weighted images in three orthogonal planes were used to guide the localization of brain areas, namely, left frontal lobe white matter (FWM) and left basal ganglia (BG) (caudate head nucleus) for acquisition of ^1^H MR spectra using single voxel MR spectroscopy. In addition, voxel was placed on the CNS lesion in subjects of Group 3. It was also ensured that the selected voxel in the FWM or BG of HIV patients with CNS lesions did not include any lesion or part of it. Single voxel ^1^H MRS spectra were acquired with a point resolved spectroscopy (PRESS) localization pulse sequence at an echo time (TE) of 35 ms and a repetition time (TR) of 2000 ms with 64 or 128 scans. The voxel size used for acquiring the MRS was 16 mm × 16 mm × 16 mm for the FWM and 12 mm × 12 mm × 12 mm to 14 mm × 14 mm × 14 mm for the BG depending upon the individual subject’s brain anatomy. The absolute concentrations of neuro-biochemicals were estimated using Linear Combination Model software (LC Model, version 6.3-1) which performs deconvolution of spectra by using a basis set of reference spectra [12]. The preprocessing of MR spectra is fully automated by LCModel software including phase, baseline and eddy current correction. Water scaling method using unsuppressed water spectrum acquired from the same voxel used for acquiring water suppressed spectrum. Basis set of spectra (provided by the vendor) for individual neuro-biochemical was used for automatic fitting routine for estimating the absolute concentration and the Cramer-Rao Lower Bounds values for each metabolite. The metabolite concentrations were reported as obtained directly from the LCModel output and were not corrected for T1 and T2 relaxation effects. This method includes correction for T2 relaxation time and the number of ^1^H nuclei contributing to the resonance peak. In the ^1^H MR spectrum obtained, the main resonance peaks identified were arising from tNAA (NAA + N-acetylaspartylglutamate, NAAG) at 2.0 ppm, tCr (Cr + phosphocreatine, PCr) at 3.0 ppm, Cho-containing compounds (tCho) at 3.2 ppm, glutamate + glutamine (Glx) at 2.2-2.4 ppm, myoinositol (mI) at 3.5 ppm and lipid/macromolecule (Lip09 + MM09) at 0.9 ppm. These resonances were analyzed to estimate the concentration (in institutional units) of respective metabolite and the Cramer-Rao lower bounds for all metabolites analyzed in the study were below 15 %.

### Statistics

Data was analysed by Stata 12 (Statacorp 4905, Lakeway Drive, College Station, Texas) and described by mean, standard deviation, frequency and percentage. Karl-Pearson/ Spearman correlation coefficient was used to assess the correlation between two continuous variables. Continuous variables were compared among the Group by one way ANOVA followed by post-hoc comparison with Bonferroni correction. p-value less than 0.05 was considered as significant.

## Results

### Demographic profile and clinical features

Mean age among three Groups was not statistically different with the mean age of Group 1 34.3 ± 12.5 years, Group 2, 37.0 ± 13.1 years and 37.3 ± 10.6 years in Group 3. 50 subjects (70.4 %) were male whereas 21 subjects (29.6 %) were female, 48 subjects (67.6 %) were married and 23 subjects (32.4 %) were unmarried (Table 1).

**Table 1:** Comparison of baseline characteristics among three groups

Variable	Healthy controls	Asymptomatic HIV	HIV with CNS lesion	Total
	Group 1	Group 2	Group 3	
	*n* = 30	*n* = 20	*n* = 21	
Gender				
Male	19	11	20	50 (70.42 %)
Female	11	09	01	21 (29.58 %)
Marital status				
Married	16	17	15	48 (67.61 %)
Unmarried	14	03	06	23 (32.39 %)
Socioeconomic status				
Upper (I)	17	02	01	20 (28.17 %)
Upper Middle (II)	06	03	02	11 (15.49 %)
Lower Middle (III)	02	05	03	10 (14.08 %)
Upper Lower (IV)	04	06	15	25 (35.21 %)
Lower (V)	01	04	00	05 (07.04 %)
High risk behavior				
None	30	00	00	30
Not specified	00	04	01	05
Heterosexual intercourse	00	13	18	31
I.V. needle sharing	00	00	02	02
Blood transfusion	00	01	00	01
Tattooing	00	01	00	01
From spouse	00	01	00	01
Presenting complaints				
None	30	00	00	30
Fever	00	11	08	19
Altered sensorium	00	00	11	11
Retrospective after spouse	00	03	00	03
Antenatal screening	00	02	00	02
Others	00	04	02	06

The mean duration between ART initiation and MRS study for Group 2 is 6.3 ± 5.9 months whereas for Group 3, most of the patients were examined within a month of ART therapy except 3 patients which were studied at 3, 38 and 144 months. However, it is difficult to know the exact time of HIV infection.

No history of high risk behavior was seen in healthy subjects; heterosexual intercourse was found to be most common mode (31 patients) of HIV transmission in Group 2 and Group 3. Other high risk behaviors include I.V. needle sharing (2 patients), blood transfusion (1 patient), and tattooing (1 patient), from affected spouse (1 patient) and in 5 HIV patients no specific etiology could be elicited on the basis of the history. The most common complaint in Group 2 was fever (55 %) followed by other causes (20 %), retrospective diagnosis after spouse (15 %) followed by during ante natal check-up (10 %). In Group 3, the most common presenting complaint was altered sensorium (52.4 %), followed by fever (30.1 %) and other causes (9.5 %) (Table 1) .There was no significant difference in pulse rate and diastolic blood pressure among three Groups studied. Systolic blood pressure was significantly lower in Group 3 compare to Group 1 and respiratory rate was significantly higher in Group 3 compared to Group 1.

### Biochemical parameters

CD4 count was significantly lower (*p*-value <0.001) in Group 3 (106.95 ± 86.94 /ul) compared to Group 2 (257.40 ± 129.09 /ul). Patients of Group 2 and Group 3 were found to have significantly lower levels of hemoglobin (*p*-value <0.0001), serum calcium (*p*-value < 0.0001), serum sodium levels (*p*-value <0.0008) and serum albumin levels (*p*-value <0.0001) compared to Group 1 (Table 2).

**Table 2:** Comparison of laboratory parameters among three groups

Variable	Healthy controls	Asymptomatic HIV	HIV with CNS lesion	*p*-value	Post-hoc comparison		
					I vs II	I vs III	II vs III
	Group 1	Group 2	Group 3				
	*n* = 30	*n* = 20	*n* = 21				
Age	34.33 ± 12.5	36.95 ± 13.1	37.29 ± 10.6	0.63			
Respiratory rate Per minute	15.8 ± 1.1	15.4 ± 1.1	16.6 ± 2.2	0.04	1.00	0.19	0.04
Systolic blood pressure in mmHg	121.67 ± 7.47	118.5 ± 7.42	112 ± 12.55	0.002	0.72	0.001	0.08
CD4 count/ul		257.40 ± 129.09	106.95 ± 86.94	0.0001			
Hemoglobin gm/dl	14.49 ± 1.57	12.46 ± 1.28	10.68 ± 2.69	0.0001	0.001	0.001	0.01
Serum Calcium Meq/L	9.16 ± 0.6	8.56 ± 0.77	8.16 ± 0.64	0.0001	0.007	0.001	0.18
Serum sodium Meg/L	140.03 ± 4.04	138.05 ± 4.51	134.76 ± 5.52	0.0008	0.43	0.001	0.72
Serum albumin gm/dl	4.56 ± 0.77	3.89 ± 0.81	3.36 ± 0.61	0.0001	0.008	0.001	0.72
Serum globulin gm/dl	2.43 ± 0.59	3 ± 0.46	3.6 ± 0.98	0.0001	0.01	0.001	0.72
LDL mg/dl	100.37 ± 15.33	103.7 ± 20.9	126.62 ± 20.9	0.0001	1	0.001	0.001

Oneway ANOVA followed by post-hoc comparison with Bonferroni correction

Serum globulin levels were significantly high (*p*-value < 0.0001) in Group 2 (3 ± 0.46 g/dl) and Group 3 (3.6 ± 0.98 g/dl) when compared to Group 1 (2.43 ± 0.59 g/dl). Although there was no significant difference in the levels of cholesterol, HDL and triglycerides but LDL level was significantly higher (*p*-value < 0.001) in Group 3 (126.62 ± 20.9 mg/dl) compared to Group 1 (100.37 ± 15.33 mg/dl) and Group 2 (103.7 ± 20.9 mg/dl) (Table 2).

### MRI and MRS data

Among 30 healthy controls, 21showed a normal MR imaging and 7 showed non-specific white matter changes. In Group 2, 13 patients showed normal MRI whereas 7 patients showed non-specific white matter changes. Group 3 is a heterogeneous group comprising patients of HIV with tuberculomas (14 patients), progressive multifocal leucoencephalopathy (4 patients), cryptococcal meningitis (2 patients) and 1 patient of suspected intracranial malignancy.

There was no significant difference in the level of tCho in FWM and BG among the three group studied. Patients of Group 3 were found to have significantly lower levels of tNAA in BG (*p*-value < 0.05). Significantly high levels of Glx (*p*-value < 0.05), tCr (*p*-value < 0.05) and Lip09 + MM09 (*p*-value < 0.04) were seen in FWM of Group 3. In BG, Glx, tCr and Lip09 + MM09 levels were not significantly different among the three groups studied (Table 3).

**Table 3:** Comparison of concentration of neuro-biochemicals among three groups

Variable	Healthy controls	Asymptomatic HIV	HIV with CNS lesion	p-value	Post-hoc comparison		
					I vs II	I vs III	II vs III
	Group 1	Group 2	Group 3				
	*n* = 30 (mmol/kg)	*n* = 20 (mmol/kg)	*n* = 21 (mmol/kg)				
tNAA							
Frontal	9.29 ± 3.11	10.67 ± 3.8	10.99 ± 4.11	0.24	0.56	0.44	1
Basal ganglia	7.31 ± 0.47	7.29 ± 0.74	6.71 ± 0.64	0.01	1	0.01	0.03
Lip09 + MM09							
FWM	5.59 ± 1.56	5.14 ± 1.79	8.69 ± 2.96	0.02	1	0.04	0.11
BG	5.87 ± 1.05	5.76 ± 1.95	5.59 ± 0.53	0.78	1	1	1
tCho							
FWM	2.08 ± 0.70	2.50 ± 0.99	2.62 ± 0.78	0.07	0.26	0.12	1
BG	1.62 ± 0.17	1.68 ± 0.28	1.56 ± 0.16	0.27	1	1	0.33
tCr							
Frontal	6.95 ± 2.56	8.9 ± 2.9	9.14 ± 3.04	0.02	0.05	0.05	1
Basal ganglia	6.95 ± 1.51	6.3 ± 1.77	6.62 ± 0.78	0.19	0.4	0.38	1
Glx (Glu + Gln)							
FWM	15.0 ± 6.06	17.08 ± 5.6	20.4 ± 7.8	0.05	0.82	0.05	0.47
BG	13.99 ± 2.89	14.12 ± 3.6	13.92 ± 1.67	0.98	1	1	1

Oneway ANOVA followed by post-hoc comparison with Bonferroni correction

**Fig. 1:**
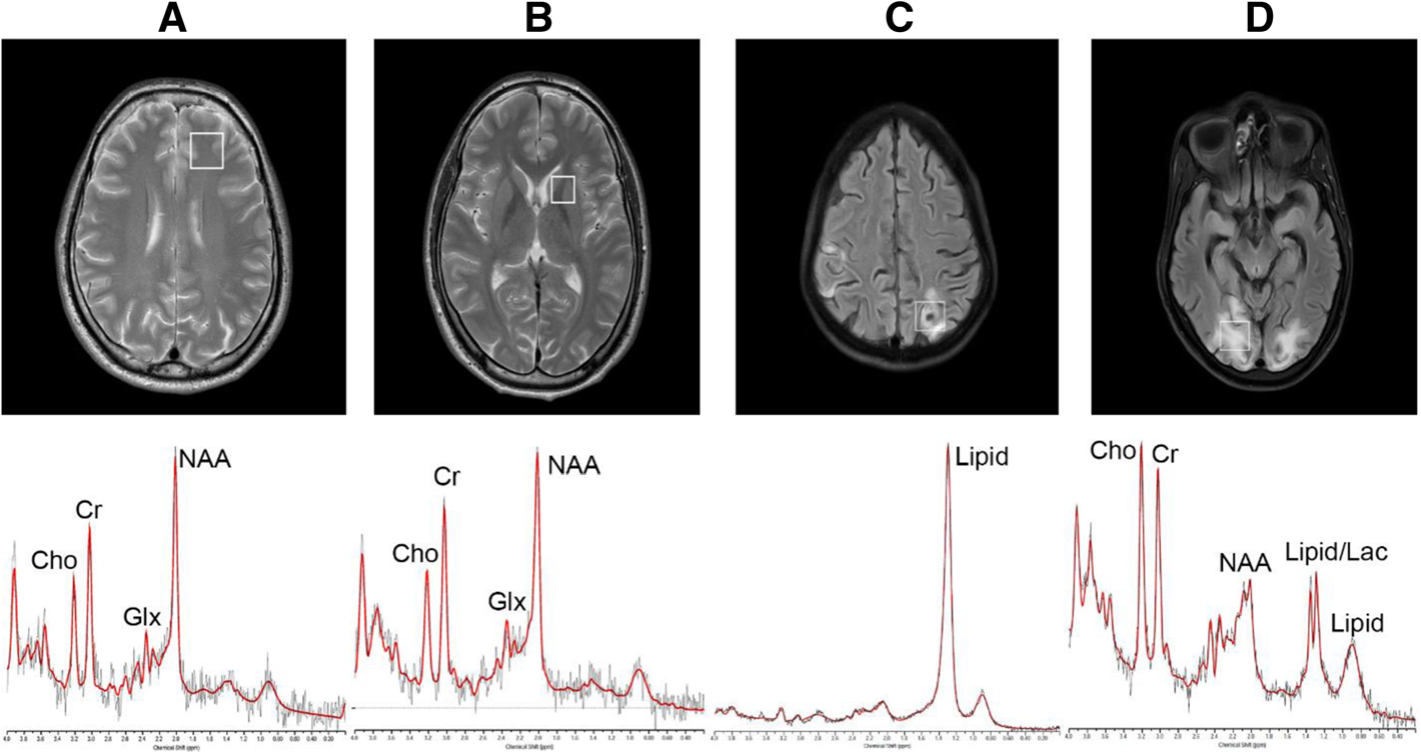
In vivo ^1^H MRS spectrum from **a** left frontal white matter and **b** left basal ganglia of a healthy control, **c** tuberculoma in an HIV patient and **d** white matter lesion from an HIV patient with Progressive Multifocal Leucoencephalopathy. Abbreviations: NAA, N-acetylaspartate; Cho, choline; Cr, creatine;Lac, Lactate; Glx, Glutamate + Glutamine

Out of 14 HIV patients with CNS tuberculomas, 5 patients (35.7 %) showed lipid peak while 3 subjects (21.4 %) showed lactate peak from the lesion. Out of 4 HIV patients with PML, one subject (25 %) showed lipid peak and two (50 %) showed lactate peak from lesion (Fig. 1). No lipid and lactate peak were observed in HIV patient with suspected malignancy and cryptococcal meningitis. We did not find any correlation of laboratory parameters namely, CD4 count, hemoglobin, fasting blood sugar, lipid profile, urea, creatinine or liver enzymes with neuro-biochemical levels.

## Discussion

The immunosuppressed state in HIV patient makes CNS vulnerable to opportunistic infection and tumors as well as susceptible to damage from HIV itself [13]. MRI of brain is a sensitive imaging modality for detection of brain abnormalities in patients infected with HIV, however conventional MRI is not always sensitive for the detection of the early stages of brain HIV infection [14]. No study till date has compared simultaneously healthy controls, asymptomatic HIV patients and HIV patients with CNS involvement by infection or malignancy. We quantified the levels of neuro-biochemical to compare with the results of previous studies.

### Clinical evaluation and laboratory findings

It is expected that during the early stages of HIV infection, there are no neurological abnormality or anatomical changes apparent on MRI. However, it is possible that there may be some molecular and metabolic changes occurring in the brain due to HIV infection. Therefore asymptomatic HIV patients (Group 2) were recruited to investigate whether the HIV infection itself has some impact on brain neuro-biocehmicals even without having overt involvement by infection or malignancy. Majority of the patients of Group 3 were critically ill and were having septicemia because of infection due to underlying immunosuppression and septic shock. Therefore these subjects showed significantly low systolic blood pressure and increased respiratory rate as a component of systemic inflammatory response syndrome (SIRS) [15]. Lower CD4 count is associated with an increased risk of opportunistic infection and increase risk of malignancy. The Swiss HIV Cohort study reported that the risk of developing opportunistic infections is increased by 2.5 if the CD4 count is between 51–200 cells/mm; this risk increases to 5.8 for counts <50 cells/mm [16]. Various studies have demonstrated that lower CD4 counts are associated with an increased prevalence of opportunistic infections [17, 18]. This explains the probable presence of various opportunistic infections like tuberculosis, PML and cryptococcal meningitis in patients of Group 3.

Anemia is a frequent complication that occurs in 20 to 80 % HIV-infected persons and is associated with faster disease progression and mortality [19]. HIV infection may lead to anemia in many ways: changes in cytokine production with subsequent effects on hematopoiesis [20] decreased erythropoietin concentrations [21], opportunistic agents such as *Mycobacterium avium* complex [22], administration of chemotherapeutic agents such as zidovudine, ganciclovir [23] and cotrimoxazole [24] and myelophthisis caused by cancers such as lymphosarcoma. Anemia has been associated with progression to AIDS and shorter survival times [25] for HIV-infected patients.

Disturbances of electrolyte metabolism and endocrine regulation like hyponatremia [26, 27], hypo- and hyperkalemia [28, 29] have been observed in HIV patients. Hypocalcaemia, defined by serum calcium level less than 8.5 mg/dl [30], could be caused by HIV infection [31]. Diarrhea is a common condition seen in HIV and diarrheal diseases could also cause hypocalcaemia through malabsorption and sepsis [32, 33]. It has long been noted that hyponatremia is associated with pulmonary or central nervous system (CNS) infections [34], gastrointestinal sodium losses [35], adrenal insufficiency [36] and renal disorders [37]. Importantly, a CNS or pulmonary infection may result in a syndrome of inappropriate secretion of antidiuretic hormone (SIADH), which plays a pivotal role in the presentation of hyponatremia [35, 38, 39]. Therefore hyponatremia can be correlated with disease progression in HIV.

Serum albumin, is a plasma protein produced by the liver, has a role in many physiologic processes, including vasodilation, endothelial cell apoptosis, and antioxidant reactions [40]. Individuals with conditions such as malnutrition, chronic inflammation, enteropathy, or liver disease can have reduced serum albumin concentrations [40]. Several studies of HIV-infected individuals have determined that hypoalbuminemia is associated with more rapid progression to AIDS and all-cause mortality in developed countries [41, 42]. Globulin levels are also increased in HIV because of coexisting or ongoing chronic inflammatory process.

### MRS findings

#### tNAA and tCr

NAA is a neuronal marker and is evenly distributed throughout the cerebral cortex. Although its exact function is not known but it is widely accepted that decreased levels of NAA is a marker for neuronal loss. NAAG is a neuron-specific dipeptide synthesized from NAA and Glu by NAAG synthase, present in brain at its highest concentration [43, 44]. NAAG and its precursor NAA are proven by immune-histo-chemistry to be present within the bodies of neuron [45]. The resonance peak of the methyl group of NAAG at 2.04 ppm strongly overlaps with methyl resonance of NAA in brain MRS. Our findings are in agreement with a previous report [46], which showed decreased tNAA levels in BG but not in FWM of acutely infected HIV patients. BG comprised of neuronal cell bodies and FWM is composed of mainly nerve fibers. The observation of decreased levels of tNAA in BG may indicate significant neuronal loss in this region compared to FWM.

In the present study, a significant increase of tCr was seen in FWM in Group 3 compared to Groups 1 and 2. This may be due to two possible factors: (i) glial cells contain higher Cr content than neurons therefore, a cell population change results in change of Cr level and, (ii) the equilibrium between PCr and Cr in creatine kinase reaction may change due to the disease progression [47]. Previous studies have shown changes in Cr levels in brain of patients with HIV infection. Ernst et al. reported decreased concentration of Cr in right basal ganglia in patients with HIV and progressive cognitive impairment with motor dysfunction [48]. In a study on ART naïve HIV patients Chang et al. reported an increase in Cr in frontal white matter and correlation with CD4 count [49]. These results suggest region specific changes in Cr metabolism in HIV patients; however, further studies are required to substantiate these findings. Moreover, most of the previous studies analyzed ratios of neuro-biochemicals with respect to Cr, assuming that the concentration of Cr do not change significantly [5–7, 50, 51]. However, it has been shown that Cr levels can increase with age and trauma as a hyperosmolar response and some brain pathologies like stroke, tumor, lymphoma, toxoplasmosis are characterized by reduced Cr [45].

### Glutamate and glutamine (Glx)

Metabolically Glu is stored as glutamine (Gln) in glial cells, and the balanced cycling between these two neuro-biochemicals is essential for normal functioning of brain cells. Astrocytes are responsible for uptake of most extracellular Glu via the high-affinity Glu transporters GLT1 and GLAST, and additionally have a vital role in preserving the low extracellular concentration of Glu needed for proper receptor-mediated functions, as well as maintaining low concentrations of extracellular Glu to prevent excite-toxicity [52, 53]. Therefore Gln is an astrocyte marker and Glu is an important neurotransmitter [45]. Glutamate release is subject to calcium regulation, and CD38 is an enzyme in astrocytes involved in intracellular calcium signal. CD38 up-regulation was demonstrated by immune-histo-chemical analysis in brain tissue from HIV encephalopathy (HIVE) patients [54]. Activated astrocytes increase CD38 expression after IL-1β treatment, and this up regulation is mediated by the MAPK and NF-kB signal cascades [55]. Thus the resulting over expression of CD38 partially occurred due to the evoked Glu release, which was in turn caused by elevated Ca^2+^ influx [56]. However, the role of increased levels of Glu in CSF or plasma as an useful indicator of HIV-associated excite-toxicity remains to be investigated. Experimental evidence suggests that excite-toxicity might play a major role in HIV-induced neuro-degeneration. Ferrase et al. showed increased Glu levels in CSF of HIV patients, compared to healthy subjects, also in patients with Alzheimer-type dementia, and patients with other neurologic disorders [9]. Increased glutamate levels in the CSF and plasma of HIV-infected patients and the glutamate levels positively correlated with the degree of dementia and brain atrophy [10]. Nonetheless, it is widely accepted that brain metabolite dysregulation occurs early in HIV infection [57] and persists in the setting of chronic and stable disease [58]. Clinical studies using ^1^H MRS to evaluate the metabolite levels in early versus chronic cognitively normal HIV+ subjects showed a reduction in the Glx levels of HIV+ subjects compared to controls [57, 59]. It should be noted that Ernst et al. [60] showed no significant increase in white matter Glu levels after adjusting p-value for multiple comparison.

The present study showed increase in Glx in FWM of HIV patients with CNS lesions compared to asymptomatic HIV patients and healthy controls. Metabolism and regulation of Glu and Gln is a complex process in patients with HIV and requires further studies to understand these changes in relation with HIV infection.

### Choline and myo-Inositol

myo-Inositol (mI) is a known glial marker. Many previous studies have shown either an increase in mI [6, 51] or no change in mI levels [7, 57]. The present study does not reflect any change in the brain mI levels. Cho is considered to be a marker of cell membrane and previous studies have reported variable results. While most of the studies showed increased Cho/Cr ratio with HIV infection [5, 50], few depicted no change in Cho/Cr ratio with HIV infection [6, 7]. We did not find any significant changes in choline levels in Group 2 and Group 3 compared to Group 1.

### Lipid and macromolecules

The present study showed significant increase in Lip09/MM09 signal at 0.9 ppm in FWM in HIV patients with CNS lesion compared to healthy subjects. Over 20 % of the dry weight of brain is lipid and these macromolecules do not appear unless some pathological process liberates the MR visible triglycerides and long chain fatty acids as seen in infection, inflammation, necrosis or stroke [45]. Salvan et al. conducted a study on HIV infected children older than 2 years using ^1^H MRS to study their cerebral metabolism and to identify metabolic profiles in relation to different stages of the disease. A significant increase of the proportion of the lipid signals (*p*-value <0.05) was found in all HIV-infected children compare to HIV negative controls. In our study, Group 3 showed increased levels of Lip09/MM09 in FWM compared to healthy controls. In a study by Roc et al., the lipid and lactate/Cr ratio was significantly elevated in HIV positive subjects irrespective of the level of neuro-cognitive impairment on the MRS study of lenticular nuclei [61].

There are some limitations of the present study; the low number of patients in Group 3 which is primarily due to reason that these patients were on life support and with altered sensorium posing a difficulty to subject them to MRS. The number of patients with tuberculomas, PML, cryptococcal meningitis and suspected intracranial malignancy were very low and hence sub-group analysis could not be carried out. Further, it is difficult to conclude with certainty if the neuro-biochemical changes seen were either caused by the varying tissue composition in different MRS voxel or the neuro-biochemical change was associated with the cell population change during the diseases progression. In MRS there is possibility of partial volume effect from different tissues, which is difficult to avoid in some anatomical areas with cuboid shaped voxel. In our study, the voxel size and location was adjusted to match the target anatomical area as accurately as possible, however, there is possibility of varying tissue composition being included in the voxel. In addition, comparatively smaller voxel size might have resulted into low signal to noise ratio in some of the spectra obtained. For example, in case of voxel localization in FWM, there is possibility of inclusion of gray matter. We have not acquired the tissue composition data for each voxel to correct for these partial volume effects. In addition, it is difficult to differentiate between lipid and lactate signal at TE of 35 ms.

## Conclusion

The results of the present study showed significant increase in Glx and tCr in FWM of HIV patients with CNS lesion compared to asymptomatic HIV patients and healthy controls. In BG, there is significant reduction in tNAA. Asymptomatic HIV patients do not have significant alteration in brain neuro-biochemicals compared to healthy controls. Our findings support excito-toxicity effect of increased Glx in HIV associated dementia and region specific metabolic changes in brain associated with HIV infection.

## Abbreviations

AIDS, Acquired immunodeficiency syndrome; ART, anti-retroviral therapy; BG, left basal ganglia caudate head nucleus; CNS, Central nervous system; Cr, Creatine; CSF, cerebrospinal fluid; ELISA, Enzyme linked immune-sorbent assay; FWM, left frontal lobe white matter; GLAST, glutamate astroglial transporter; GLT-1, glutamate transporter type 1; Glx, Glutamate and glutamine; HAND, HIV associated neuro degeneration; HDL, high density lipoprotein; HIV, Human immunodeficiency virus; HIVE, HIV associated encephalopathy; LDL, low density lipoprotein; MAPK, mitogen activated protein kinase; mI, myo-Inositol; MRI, magnetic resonance imaging; MRS, magnetic resonance spectroscopy; NAA, N-acetylaspartate; NAAG, N-acetylaspartylglutamate; NF-kB, nuclear factor –kB; PML, progressive multifocal leucoencephalopathy; tCho, Choline and other choline containing compounds
